# Health professionals and patients’ perspectives on person-centred maternal and child healthcare in Burkina Faso

**DOI:** 10.1371/journal.pone.0230340

**Published:** 2020-04-01

**Authors:** Thècle Twungubumwe, Mylène Tantchou Dipankui, Landry Traoré, Johanne Ouédraogo, Seydou Barro, Josette Castel, Isabelle Savard, Marlyse Mbakop Nguebou, Jean Ramdé, André Côté, Judith Lapierre, Ruth Ndjaboue, Maman Joyce Dogba

**Affiliations:** 1 Department of Family and Emergency Medicine, Faculty of Medicine, Université Laval, Québec, Canada; 2 Regional Health Department / Ministry of Health, Ouagadougou, Burkina Faso; 3 Vice Dean for Social Responsibility, Faculty of Medicine, Université Laval, Québec, Canada; 4 Université TÉLUQ, Québec, Canada; 5 Faculty of Education, Université Laval, Québec, Canada; 6 Faculty of Administration, Université Laval, Québec, Canada; 7 Faculty of Nursing, Université Laval, Québec, Canada; 8 Centre for Research on Primary Care and Services, Université Laval, Québec, Canada; University of Ghana College of Health Sciences, GHANA

## Abstract

**Context:**

The person-centred approach (PCA) is a promising avenue for care improvement. However, health professionals in Burkina Faso (hereafter referred to as caregivers) seem unprepared for taking into consideration patients’ preferences and values in the context of healthcare provision.

**Objective:**

To understand the meaning attributed to PCA in the Burkina Faso context of care and to identify the challenges related to its adoption from the perspective of caregivers and women service users (hereafter referred to as patients).

**Methods:**

An ethnographic qualitative research design was used in this study. We conducted 31 semi-directed interviews with caregivers and patients from Koudougou (Burkina Faso) healthcare facilities. We also carried out direct observation of consultations. Data thematic analyses are based on the person-centred approach analysis framework.

**Results:**

According to the caregivers and patients interviewed, the PCA in maternal and child healthcare in Burkina Faso includes the following five components used in our analytical framework: i) pregnancy follow-up consultations extend beyond examining physical health issues (biopsychosocial component), ii) healthcare professionals’ mood affects the caregiver-patient relationship as well as care delivery (the healthcare professional as a person), iii) patients expect to be well received, listened to, and respected (the patient as a person), iv) healthcare professionals first acknowledge that both themselves and patients have power, rights but also responsibilities (sharing power, rights and responsibilities of professionals and patients), and v) healthcare professionals who are open to involving patients in decision-making about their care and patients asking to have a say in the organization of services (therapeutic alliance). Implementing each of these themes comes with challenges, such as i) talking about health problems in the presence of other women, especially those related to sexuality, even though they are common to parturient women (biopsychosocial component); ii) offering psychotherapy to healthcare professionals (healthcare professional as a person); iii) taking into consideration patients’ cultural and linguistic differences (the patient as a person); iv) raising awareness among patients about their right to ask questions and healthcare professionals’ duty to answer them (sharing power, and rights and responsibilities of professionals and patients); v) accepting the presence of birth attendants while avoiding traditional practices that are contrary to scientific recommendations (therapeutic alliance).

**Conclusion:**

Despite some context-specific particularities, the PCA is not new in the context of health care in Burkina Faso. However, its implementation can pose a number of challenges. There is a need to train healthcare professionals with a view to being sensitive to these particularities. This may also require organizational adjustments so as to create the physical and sociocultural environments that are conducive to taking into account the patient’s perspective.

## Introduction

In 2010, the maternal mortality rate in Burkina Faso was estimated at 341 deaths per 100,000 births[1]with 80% of deaths being attributable to obstetric causes[2]. The infant and child mortality rate is also very high (probability of dying before the age of five), i.e. one out of eight[1]. These high rates are the result of difficult access to care, as well as poor use of existing services. For example, coverage of the first prenatal consultation (PNC-1) was at 95% in 2010, but only 34% of pregnant women in Burkina Faso had four prenatal visits as recommended by the World Health Organization[3]. Poor use of maternal health services is due, among other things, to the poor quality of care imposed on patients by public healthcare professionals[4, 5], such as hostility, intimidation, disrespect and even physical and verbal abuse[4, 6–9], caregiver indifference to the particularities of each patient, the lack of intimacy, absence of consent, and lack of communication between patient and caregiver[10]. These behaviours are sometimes attributed to work overload, shortage of staff, lack of professional recognition, poor accountability framework, lack of support for leaders, and an organizational culture that encourages this type of behaviour [11, 12]. The result is the abandonment of public maternal health services in favour of self-medication (43.2%) and traditional healers (11.4%), who are more attentive to women’s needs[4]. Research data shows that women choose to use healthcare services when caregivers are attentive to their preferences, values, and perceptions [13, 14]. Unfortunately, most healthcare professionals in Burkina Faso are not trained in this approach to healthcare delivery. Their initial training is mostly focused on the biomedical perspective that positions them as experts and tends to devalue the patient’s opinion [4, 15, 16]. In this context, it is therefore important to introduce holistic approaches to care, integrating patients’ values and to train health professionals within a participatory approach to interrelational skills. This approach, referred to as “patient-centred” or “person-centred” approach (PCA), is the main focus of a training program developed in the context of the second phase of the *Maternal and Child Health Improvement Program (MACHIP 2)* in Burkina Faso.

PCA can be defined as care aligned with patients’ values, their preferences and their desires. Its five fundamental principles, described further in this article include: 1) a biopsychosocial perspective, 2) power and responsibility sharing between health professional and patient, 3) the physician and caregivers as people, 4) the patient as a person, expert, resource, and partner, and 5) the therapeutic alliance. Implementing PCA in care will require from caregivers’ competences in the areas of communication (e.g. information sharing and compassion), health promotion and partnership (ranging from informing patients to fully involving them in all decisions about their care)[17, 18].

As of February 2017, to our knowledge, there is no existing culturally validated training program on the subject in Burkina Faso. In fact, most of the literature on the topic focuses on developed countries. Consequently, we are unable to identify the PCA concepts that would be best adapted to care in the Burkina Faso context. In addition, we have very little information on what it means to involve patients in their own care and health in Burkina Faso, i.e. women and children, in terms of feasibility and local understanding. They live in a social, political, and economic context that is unfavourable to them (social norm, weak political and legal framework), marginalizes their roles, and barely promotes their emancipation. Furthermore, their opinion is hardly ever considered in professional healthcare training. The data collected in this article would be the baseline for the production of a culturally adapted training program for the MACHP 2. Furthermore, it seems important to understand the meaning given to PCA in the context of care in Burkina Faso and to determine how PCA concepts can be integrated into maternal and child health professionals’ training. This article aimed to understand what PCA means and what challenges its adoption entails from the perspective of professionals and women service users. More specifically, our research questions are formulated as follows: 1) What does PCA mean in the context of care in Burkina Faso from the perspective of professionals and patients? 2) What are the obstacles to adopting the PCA in the context of care? 3) How to effectively integrate the concept of PCA in the training for maternal and child health professionals’ in the context of the MACHIP 2 project?

## Material and methods

### Research design and context

This study is based on an ethnographic qualitative research design [19]. It was carried out in the town of Koudougou (centre-west region of Burkina Faso), headquarter town of the Koudougou health district, which is also where the MACHIP 2 project was launched, hence the symbolic choice of this place as the main site for the study. The Koudougou health district has a surface area of 1,949 km^2^ and a population of approximately 384,340 as of 2017, living in six rural communities and one urban community, including 91 administrative villages [20]. The population is mainly composed of the Mossi ethnic group, but other groups are also represented, such as the Fula, Gurunsi, Samo, Wala, and Dagara peoples [21]. In 2015, there were 43 health and social promotion centres (HSPC), three medical centres with or without a surgical unit (MC/MCS) and one regional hospital (RH) [20].

## Analytical framework

**Fig 1:**
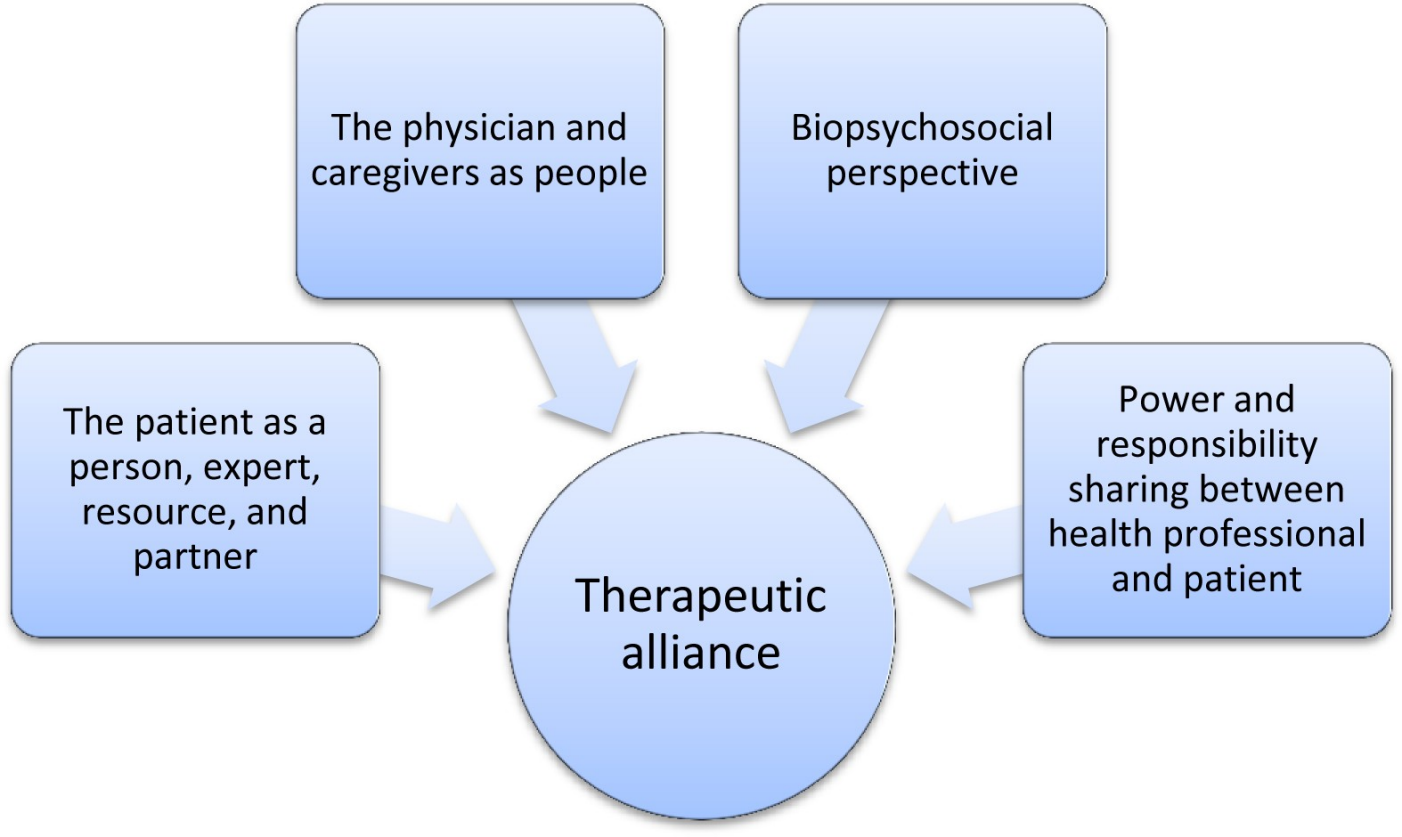
Analytical framework for the study (Adapted from Barro, 2012).

In order to tackle our research questions, we adapted the conceptual framework developed by Barro (2012). We believe that this framework is appropriate because 1) it identifies the key dimensions of PCA based on pioneering work in the area [22, 23], and 2) it has previously been used in a research project in Burkina Faso. However, we adapted it by broadening the concept of the patient as a person to include the patient as an expert, resource, and partner. According to our analytical framework, the person-centred approach has five key components: 1) a biopsychosocial perspective, 2) power and responsibility sharing between health professional and patient, 3) the physician and caregivers as people, 4) the patient as a person, expert, resource, and partner; these four components enable the fifth, which is the therapeutic alliance (Fig 1).

Components are summarized in Table 1 below.

**Table 1:** PCA components.

Components	Definitions
Biopsychosocial perspective	Care for the patient or parturient woman within a holistic perspective, i.e. beyond physical health.
Health professional as a person	The feelings experienced by professionals and how they affect the quality of their work and their relationship to patients.
Patient as a person (expert, resource, and partner)	Consideration for the patient/parturient woman as a human with a capacity to make choices and have feelings. The patient/parturient woman has an experiential knowledge, which contributes to ensuring effective care that meets her needs.
Power sharing, and rights and responsibilities of health professionals and patients	Existence of relationships of dependency and autonomy within the care space. Caregivers use information resources–their expertise–to control parturient women while parturient women use the same resources–information drawn from social media and so on to get a degree of independence–flexibility–in adopting the recommended behaviour.
Therapeutic alliance	The decision-making process concerning care, in which health professionals and patients are involved jointly.

## Recruitment of participants

The population for this study was composed of women from the centre-west region of Burkina Faso and health professionals, including physicians, nurses, state midwives, and auxiliary birth attendants. In order to be included in the study, patients had to be between 18 and 50 years of age, have visited a HSPC, a MC/MCS, or a RH within the MACHIP 2 project area over the six months prior to the study, and accept to participate. Women who visited a healthcare facility outside the area covered by the MACHIP 2 project were excluded from the study. Concerning health professionals, inclusion criteria were practicing as a midwife, nurse, or auxiliary birth attendant; providing care to women and children; at a HSPC, MC/MCS, or RH within the MACHIP 2 project area; and accepting to participate in the study. Those offering care to women and children but not employed at a healthcare facility within the area covered by the MACHIP 2 project, and those who had received medical training abroad, in Europe or North America, were excluded from the study.

Health professionals were recruited in stages with the assistance of head nurses: 1) presenting study objectives to professionals; 2) appointment scheduling with health professionals from HSPC, MC/MCS, or RH in Koudougou who were interested in participating in the study; 3) interviews conducted at the time of meeting with professionals from the Poa and Ramongo healthcare area who were interested in participating in the study. Patient recruitment was also done with the help of health professionals who recommended women to the research student at the end of pre- or post-natal consultations. The research student who also is the first author of this article was at the time of this study a public health graduate student under the supervision of the corresponding author. She explained what the study was about and made interview appointments with those who expressed interest in participating. She recruited on average three women and two health professionals per healthcare facility with a view to achieving a diversified sample.

## Data collection

Data collection was carried out between November 2017 and May 2018. Interviews, discussions, and field notes were the main tools used to collect data. Thirty-five semi-structured interviews, and approximately 275 hours of direct observation during consultations and informal conversation with health workers and women. All interviews were conducted in person by two team members (TT and TL). Two incomplete interviews and two unusable interviews (technical problem) were excluded from analysis. In total, we conducted 31 interviews (19 with patients and 12 with health professionals) of approximately 45 minutes each. Fourteen of the 31 interviews were done in Moré (local Mossi language) with the help of an interpreter. We also held two group discussions, one with professionals, the other with patients. At the end of each interview, a questionnaire was completed by respondents for the purpose of collecting sociodemographic data. Finally, we kept a field diary with our observations and thoughts throughout the process.

Interview guides (one for the patients and one for the professionals) were created by the research team (See S1 File) on the basis of our analytical framework and tested before the start of collection. They were further refined after the first interviews. We adjusted questions to cover the entire pregnancy and delivery experience. We therefore had to find women who had already given birth to interview. The interview chart included a section on each component of the conceptual framework (biopsychosocial perspective and therapeutic alliance, for example) and a general question relating to obstacles and facilitators of the person-centred approach in the Burkina Faso context. Interviews took place at the location and time that best suited the interviewee. They were audio-recorded and their content transcribed in the form of a verbatim record.

## Data analysis

Transcripts were imported into the N’Vivo12 software. A thematic analysis[24] based on the study frame of reference was carried out following the method described by Miles and Huberman [25] including coding, organization, and establishing a relationship. TT, TL, and TDM independently coded four interviews, following which a team meeting was convened under DMJ’s supervision for the purpose of establishing a consensus with a view to final coding. The coding of the remaining interviews was carried out by TT. An initial list of emerging themes was drawn up. All interviews coded by TT, TL, and TDM were merged in order to measure their correspondence in relation to themes. TT then drew up a list of the 11 most coded themes and TDM put them in relationship to one another and reworded them based on analysis framework components. This mixed inductive and deductive approach was aimed at letting emerge themes that weren’t already included in the study analytical framework. Finally, field notes were used to validate, complement, and better explain the context of the information collected during interviews.

## Ethical considerations

This project has been approved by the Université Laval research ethics committee (**N**^**o**^**2017-302/21-03-2018)** and the research ethics committee of the Ministry of Health of Burkina Faso (**N°2018-02-020)**. All participants were informed of their rights and their verbal consent was obtained. The interpreter signed a confidentiality form.

## Results

### Sociodemographic characteristics of participants

Aged between 18 and 36, most 18-year-old patients belonged to the Mossi ethnic group. Four (4) of them spoke a language other than French or Moré. All patients except one had at least one living child. Three (3) patients were part of a polygamous household (as the second wife); seven patients were unemployed; one patient had a university degree.

Nine of the 11 health professionals were women. They all spoke French and ten of them also spoke Moré. Eleven were married or in a de facto marriage and two had a university degree. Professionals were aged between 32 and 53. The sociodemographic characteristics of patients and professionals are summarized in Table 2 and Table 3.

**Table 2:** Sociodemographic profile of patients/parturient women.

	Total N = 18
Age, μ (min, max)	28 years (18, 36)
Sex	
Female	18 (100%)
Spoken language	
Moré only	7 (38%)
Moré/French	7 (38%)
Moré/French/Dioula	1 (6%)
Moré/Dioula	2 (11%)
French/Lele/Gurunsi	1 (6%)
Education	
None	5 (28%)
Primary school	8 (44%)
Secondary school	4 (22%)
College level	0 (0%)
University level	1 (6%)
Employment	
None	7 (38%)
Farmer	1 (6%)
Seamstress	3 (17%)
Saleswoman	4 (22%)
Teacher	2 (11%)
Marital status	
Polygamous	3 (17%)
Married/Common law	15 (83%)

**Table 3:** Sociodemographic profile of health professionals.

	Total N = 11
Age, μ (min, max)	39 years (32, 53)
Sex	
Male	2 (18%)
Female	9 (82%)
Spoken language	
French only	1 (9%)
Moré/ French	3 (27%)
Moré/ French and other	7 (64%)
Education	
Primary school	1 (89%)
Secondary school	4 (36%)
College level	4 (36%)
University level	2 (18%)
Employment	
Auxiliary birth attendant	5 (45%)
State midwife	4 (36%)
Male state midwife	2 (18%)
Marital status	
Polygamous	1 (9%)
Married/Common law	10 (91%)

In the following section we first present the five components of our analytical framework. For each component, we describe the themes covered, the meaning within which it is used in our study (Question 1), and then identify the challenges related to its implementation in this context (Question 2). Where applicable, we proceed to describe additional concepts or components emerging from our analyses. Finally, we discuss the suggested solutions for integrating these components in the training of professionals (Question 3).

### I. Biopsychosocial perspective

#### Pregnancy follow-up consultations extend beyond physical health problems

Our findings suggest that most professionals endeavour to offer holistic care: professionals state that they encourage them throughout their care trajectory to express and share their concerns. In addition, according to our observations, during discussions preceding prenatal consultations, women expressed their concerns in relation to their health, the care they received, as well as their everyday lives.

Before prenatal consultations, we have listening and discussion sessions. We talk about hygiene, family planning, and preparing for delivery. Participant 4 –Health worker

Prenatal consultations (PNC) are not like they used to be. We have to find out concretely what the problem is. They are not limited to a physical exam. […] I am easily able to get the person to open up and talk. Therefore, we reassure them, we can request an ultrasound to dissipate their worry; regarding nutrition, they ask what they should eat and during consultations I address this too, as well as personal hygiene, even sexual hygiene, pregnancy is not a contraindication. […]. During questions, we identify complaints, fears [that women might have], we try to reassure them and finally we do our examination on the basis of complaints. Participant 11 –Health worker

Despite these exchanges, the challenge related to taking into account this component of the PCA might be the reluctance to address health problems in the presence of other women, although they are common to parturient women, in particular those related to sexuality.

Ah! As women, there may be something you want to ask, but you’re embarrassed. […] for example, if there is something in your genitals, you want to ask if there’s something but isn’t it embarrassing? It is embarrassing to ask! Participant 2 –Parturient woman

### II. The health professional as a person

#### Health professionals’ mood affects their relationship with patients as well as care delivery

Both professionals and patients recognize that caregivers’ moods might affect the care provided to parturient women. The latter sometimes feel reassured, heard, and supported: “I liked the way I was welcomed. I felt a great deal of consolation [when I lost my baby].” Participant 13 –parturient woman. However, cases of verbal and physical aggressiveness have also been reported and attributed by patients to the professionals’ mood, which varies with their workload, tension within the caregiving team, or family problems of their own.

Sometimes you go there, and there’s a lot of work, or there has been a misunderstanding between them, they bug you and mistreat you when they talk to you. Participant 7 –Parturient woman

[…] sometimes you come and you’re so glad. Instead of a 10-minute discussion, you go on for 15 to 30 minutes and people find that you’re taking your time outside. Participant 4 –Health worker

This health professional describes how a problem at home upset her work and greatly impacted her mood, making her impatient.

I remember …anyway, I had a problem at home and then.. well, when I am on duty, I don’t even know …it’s as though I am not there. When …even when I’m working, I’m distracted, it wasn’t working at any rate. It didn’t work. Thank God, they say “first things first.” It passed. Now things are all right. When I am on duty I know I’m on duty, you see, this is my experience. I don’t know if you’re satisfied with this answer. […] hum, when it’s like this, you’re here, you can’t talk to the women. You look like you’re angry with everybody. Discussion doesn’t work. Sometimes you can even snap, “Take this off quickly! Hurry!,” well …this can happen …it’s human! That’s it. What happens is that you’re not as gentle with clients. Participant 8 –Health worker

Another health professional explained how tension among colleagues can interfere with the quality of care because they don’t request help from one another in these situations.

[…] oftentimes moods also affect the care provided to patients. Even among colleagues, even though we’re supposed to be working. When you work in a team and we’re not on good terms, because of the conflict you can’t get the others’ opinion. You try to deal with the woman on your own. Sometimes it doesn’t work, you can make a mistake. I have witnessed this.

Participant 13 –Health worker

Work overload can influence the health professional’s mood, because of the ensuing fatigue of being back and forth between consultations and births.

If you’re seeing a woman for a PNC and another one goes into labour [at the same time], this can really affect you [your mood], it affects me specifically. You’re back and forth, it’s tiresome, and again to write down things, for a consultation, really it has an impact [on your mood]. Participant 2 –Health worker

The challenge of taking into consideration this PCA component suggests the use of intervention specialists of human behaviour and interpersonal relationships, in this case psychologists, with health professionals.

We health workers also need a psychologist […] to whom we can talk about our problems. This can help us with mood management. Health worker–Discussion group with health professionals

### III. The patient as a person

#### Parturient women ask to be well received, listened to, and respected

Our results suggest that it is important to consider patients as “persons,” as “humans,” and consequently, that they should be well received, listened to, and respected in order to achieve better results in terms of health, as asserted by both professionals and patients in the following excerpts:

When you come here and you’re respected, they take care of you, it is really soothing even though you’re sick. It gives you courage (laughs). Feeling that you’re welcome is also something. It makes you feel good. Participant 1- Group discussion with patients

Welcoming someone here starts with a greeting. A greeting to the woman, how is she doing? How is her family? If she says that she is well, then we ask her what brings her to our HSPC. This is how it is. Participant 7 –Health worker

One patient describes her experience of being listened to and supported by the midwife when her child was not well, in particular the health worker accompanying her to the pediatrician and doing a personalized follow-up. The patient felt valued thanks to the special attention that she received from the midwife.

I know that since my little one was born, on February 5, we’ve never been able to find sleep. All she does is cry, all the time. So I went there and I explained that the child doesn’t sleep. The woman who was with me the day of the delivery was there, she looked at the baby like this, lifted her foot and said, “look how much weight she has lost! If you don’t take better care of her, she’ll die!” […] The other midwife followed me. She said, “Madam, I’ll go with you, there is a pediatrician at the CM (medical centre).” And she came with me to see the pediatrician. She explained [the problem] to the pediatrician. […] one day […] during the night, she called: “how are you? Do you remember me?” I recognized the voice. “And the baby, how is she?” “She’s all right,” I said, “thank you for checking on me. […] It’s reassuring.” Participant 2 –Discussion group with patients

Even when we were at the pediatric unit, one [health worker] came every morning. A trainee. Every morning she woke up at 6 and came knocking on the door. She came and greeted all the moms. “Did your baby sleep well? How did it go?” Every morning, that was her job. It made us feel reassured.” Participant 2 –Discussion group with patients

Another patient explains that respect and active listening are an important part of care, because if the health professional listens to the patient, everything goes well.

Above all respect and listening. Respect and listening. To care for patients, one must listen to them carefully. If the person is afraid…Fear, you know…if health workers could truly respect patients and listen to what they have to say, things would change. Regardless of who the person is, she must be respected and listened to. If you listen to her and you respect her now, the rest will follow.

Participant 1- Discussion group with patients

This point was supported by one of the health professionals who underscored the importance of listening attentively to patients during the labour. The frustration experienced by the mother can affect the pushing.

You might tell a woman to push and she tries but it doesn’t work. Or she doesn’t know. You must listen to her because she might want to talk. Don’t tell her to be quiet. No, listen to her. Maybe she wants to tell you that she is unable to do what you’re asking her to do. You can help her to understand what you’re asking her to do and she will try. Participant 6 –Health worker

Yes, yes, even the training that we just did […]. There was a part on compassionate care. In other words, how to cajole the women from the time she steps inside your centre until the delivery. Especially when she’s in the delivery room and there is pain, how to rock her. Because when a woman is frustrated, it affects the pushing. Participant 6 –Health worker

The patient explains that a good interaction between the health professional and the patient requires mutual respect, not threats.

It can be an obstacle, but it depends on the way the patient is spoken to. Everything depends. If you’re gentle, respectful, the person will understand you. But if you take her like this, you can see he’s threatening you or something…then you won’t understand each other. But if you respect the person, you’re gentle with her, even if the person is agitated, he will […] listen to you and accept what you’re proposing. Yes. It all depends. Participant 1 –Group discussion with patients

The challenges of implementing this component of the PCA could include cultural and linguistic differences.

Communication often poses a problem. For example, I don’t understand Gurunsi or Lele, and often women don’t speak French, Moré, or Dioula or barely get by, and in this case communication is a problem. […]. During the examination, I won’t be able to explain what the labour process is like […]. Participant 11 –Health worker

[…] I often run into difficulty because I am not Mossi. I can’t explain exactly what I want to the women. I don’t understand Moré very well. Oftentimes, when we call in a third person to explain […] we’re not sure that this person will pass on exactly what you want to say. Participant 7 –Health worker

[…] certain [health workers] answer [my questions], but some don’t understand Moré. Participant 18 –Parturient woman

One of the health professionals suggested that cultural and ethnic differences make care difficult, because in some cultures, women don’t tell you directly what their symptoms are. You have to use your imagination to determine and treat the symptoms.

For example, there are areas of… Dédougou with a large Fula population. You can’t…take the first thing the woman says. If she tells you that she has a headache…this is actually not true…maybe she has stomach ache, you have to imagine and you have to do your best to find out what the issue is in order to treat it. Participant 10 –Health worker

Finally, health professionals noted that “the person’s” (i.e. the patient), personality and life experience influenced the caregiver-patient relationship which created a challenge. For example, a woman who tended to be “too submissive” or a victim of domestic abuse would be more reluctant to talking with health professionals than an educated woman or one who lived in an urban area, as they would more easily understand professionals.

Well, you know, what I described, it’s not fear, it’s the living environment itself…[…] of women who are subordinate within the home; really, when she comes here too, you can feel that she doesn’t like to talk. She doesn’t like to talk! It makes a difference […]. Participant 1 –Health worker

If you take women living in cities, when you come to the consultation, they communicate correctly, even well…Compared to someone who comes from the bush, you talk to him and he just looks at you; with this kind of patient, it’s a little difficult (laughs) to talk about the caregiver-patient relationship. With patients from big cities, there is no problem, they understand. Health worker–Discussion group with a health professional

### IV. Power sharing, and rights and responsibilities of health professionals and patients

#### Caregivers must demonstrate their expertise in dealing with educated patients who take part in decision-making, or conversely, have resigned themselves

Our findings suggest that there are relationships of dependency and independence within the healthcare space. On the one hand, professionals must demonstrate that they are holders of expert knowledge in relation to care, especially in dealing with informed patients.

Some are well informed and knowledgeable; they come at least to see us. In this case, you have to even prove your expertise that you are the health worker, that you are…you are the provider. You also have to prove to the patient that you were trained to provide care. Participant 10 –Health worker

On the other hand, patients who state that they have no medical knowledge let professionals make the final decision regarding care and resign themselves to follow their instructions.

We don’t know anything about this…for me, midwives are the ones who should make decisions about childbirth. […] Anything they say is […] right. Participant 16 –Parturient woman

If you go, you go with the patient, you explain the problem. If you want to ask them whether they can prescribe a given product, they’ll tell you that since you know this, you should have stayed home and treated your child yourself…The patient does not have the right to say anything over there. If you know this, stay home. If you come, you have to be careful with your words. Sometimes it is even better to stay silent and let them take care of things. Participant 2 –Discussion group with parturient women

The challenges of implementing this PCA component might include patients’ ignorance about their rights and health professionals’ responsibilities. In fact, they also seem to be unaware of their right to expression and the professionals’ duty to answer their questions.

Patients don’t have the right to say anything over there. If you know this, you should have stayed home. If you come, you have to be careful with your words. Sometimes it is even better to stay silent and let them take care of things. Participant 1 –Discussion group with parturient women

If they don’t give us an opportunity to ask questions, we’d be afraid. We don’t know whether they would be willing to answer […]. Participant 12 –Parturient woman

### V. Therapeutic alliance

#### Healthcare professionals are open to involving patients in making decisions about the patients’ care

Healthcare professionals underscored the importance of involving the women in decision-making related to their care, because they are the ones who are directly concerned and their collaboration is essential to effective care.

It works well when we try to involve the woman. She is the person concerned. So if we explain the dangers to her, it works. Care at least, works quite well. Participant 13 –Health worker

It must help, I think to myself, when the woman is involved, because…a woman can be told not to do heavy chores, or not to consume certain things. We can tell her to come get her blood pressure checked every second day or every day. It helps and it can help us too…I find that this is good, especially for the woman herself. When she’s aware, she can be careful. There. She can be careful. Participant 8 –Health worker

Professionals place emphasis on what it takes to successfully achieve therapeutic alliance. They underscore the importance of creating a relationship of trust and even friendship with the patient, to explain her condition and the therapeutic intervention to her in accessible terms.

Some people, they will only talk to a health worker that they know personally. […] Then, you can say anything. When you don’t know the person, it’s a little complicated, a bit difficult. So often, if you’re sick too, you want to look for someone you know in order to better express yourself. Participant 1 –Group discussion with parturient women

[…] I think nodules are a medical term. […] the women don’t know what nodules are. But if you say “pimple,” they know what pimples are […]. Participant 7 –Health worker

[…] When doing treatment, it is necessary to explain to the woman what we’re doing, because she doesn’t know what is going on. For example, I’ll give an example. Say you bring her into the consultation room, you want to do a gynecological exam. You tell her to lie down, “take your clothes off and lie down” without her knowing what you’re looking to do, they often become frustrated. Participant 7 –Health worker

#### Patients want to have a say in health service organization

Patients deplore the fact that a number of decisions, in particular those concerning health service organizations such as the number and frequency of PNC, are taken without allowing them to have their say in the matter. It becomes difficult for them to adjust to health centre activity schedules because going there does not guarantee that they will be seen by a health professional.

When you want to go, you have to get there early. If you go early, you’re seen rapidly, but if you go late and they’ve reached the number of people they can take in, you have to go back the following day. Participant 6 –Parturient woman

You say no, I’ve come for the PNC. They’ll tell you, “get up, you have to come back such other day”. So…And there are others, they’ll have to leave…3 km to get there. And if you go, you’re not taken, you have to go back again. Participant 1 –Group discussion with parturient women

Health professionals stipulate that they sometimes adapt their recommendations to the patient’s lifestyle despite the resulting risks.

[…] because some women will tell you, about taking iron for example, that they drink dolo [alcohol] but that they often forget […]. This is not recommended but we have no choice […], we have to tell them to cut down on their consumption. But to stop altogether is impossible. She’ll tell you what would she eat if she stopped? Participant 7 –Health worker

This kind of flexibility of health professionals sometimes makes it possible to improve service organization as reported below:

This is a patient working with the local bank, who came and saw that the women were sitting on the floor. She came, she was pregnant, and she said that she would go see the bank manager since she worked there, and they helped us by providing these benches. So we must be open… Participant 10 –Health worker

However, professionals note a significant challenge in relation to this alliance, namely accepting the presence of birth attendants in the delivery room while also avoiding the traditional practices that they apply, that are contrary to scientific recommendations. In fact, health professionals recognize that their presence in the delivery room is useful. They encourage parturient women during labour and help with small tasks.

The training we received in school, they told us to keep birth attendants in the delivery room. She’s there, if we need something, she’ll help, to assist until the delivery. […] The loincloths are there, the birth attendant is behind, she follows everything. Participant 1 –Health worker

Some professionals seem more favourable to their presence in the delivery room than others, because it would infringe on the confidentiality of their practice.

Some of them, they come in and watch then go talk about what they’ve seen as there’s nakedness, she can go out and tell somebody what happened, and there are others, well! It’s human, we can make mistakes in our interventions. There are patients who follow, if you let them in and they see this, the next day word is out. You didn’t do it intentionally but they, they will go out and talk. Participant 13 –Health worker

Health worker: there are others…me in particular…When I first started practicing, I used to let them come in. […] because in almost every family there is a woman who does delivery. So they watch what you do and then they will try to copy. And then try delivering at home. This is my understanding anyway. Participant 8 –Health worker

In addition, these birth attendants sometimes encourage practices that are dangerous for the child, which can be a source of frustration for the health professional.

For example, some older women, when you wait for the head to come out before you ease the baby out, they find that no, according to their practices, they push on the uterus to pushout the baby. We tell them no, this is not how it is done. They say that if you don’t do that, the baby will only come out a little or it won’t go through. And if by chance dystocia occurs and you have to refer, she’ll say that it’s because you don’t know how to do things, that you should push in order for the baby to come out. Participant 4 –Health worker

If birth attendants come in to threaten you, you see that this is not easy. You do your job and somebody comes and tells you that you don’t know how to do your job and wants to show you? You’re not going to accept that.: If that’s the case, here in Burkina Faso, we don’t agree. Participant 10 –Health worker

Finally, certain patients believe that birth attendants should be allowed to make practical interventions when the health professional refuses to carry them out.

When the labour started and we arrived, the birth attendant delivered my baby because the nurse refused to do it. If I hadn’t already had a child, I would have lost the baby. Participant 16 –Parturient woman

### VI. Other PCA components

In this section, we lay out some other themes that are not part of our analytical framework but that health professionals and patients found important to consider in the implementation of the PCA.

#### Violence against patients in health care settings is tolerated and justified

Whereas a number of health professionals and patients deplore the acts of aggressiveness and violence that patients have been victims of, some professionals justify them, evoking the well-being of both mother and child and the imperative to administer quality treatment or risk losing their lives.

I’m not going to lie, I hit a girl once, but she was young, because she had performed a clandestine abortion at home. When she came and we examined her, the exam was incomplete because we had to lie her down and palpate. We tried everything, she was refusing and when she expelled, we had to take out the blood clots. She continued to resist and I threatened her and hit her a bit. I’m sorry to say! But that day, she had provoked the miscarriage herself and this resulted in complications. She had to let us treat her. I don’t regret it because it was normal. Participant 11 –Health worker

Well…Hitting there…or even hitting…It happens that we hit. When you’re alone, you have to get on with it. You’ve prepared everything, everything, you do this, you do that […] to be able to perform the delivery. And to your dismay, she crosses her legs. It can be systematic. You can yell at her, like you can hit her foot. This is what people are criticizing but […] as a health worker, I don’t see how this is bad. In a way, it’s to save a life that we do this […] because when a child dies, that too is a problem […]. Participant 8 –Health worker

Other health professionals deplore this violence, but they accept it out of solidarity with colleagues.

[…] We even often yell at women during PNC. You can see that the scolding isn’t worth it, but she is nevertheless scolded. But often, to avoid frustrating the person you can use other means to defend a colleague. You calm down the woman and it passes. Participant 7 –Health worker

#### Male involvement in care can contribute to the mother and the child’s health

A number of the challenges to PCA mentioned by professionals and patients were related to gender. In fact, our results have brought to light the place of men in maternal and child care. Women are said to better interact with male health professionals (male nurses) as stated by this parturient mother.

Well, it’s hard with midwives, they talk to you as though you were a kid, others know that you’re not feeling well but they tell you that they’re tired and ask you to come back the following day, women are complicated. […] they know women’s pain and yet, they are worse than men. Participant 14 –Parturient woman

However, some are reluctant to consulting male health professionals because of religious principles.

[…] We’re lucky here, the people who come here generally accept to be examined; it’s rare but I saw a woman who was religious and she didn’t want to be examined by a man, and I got it, I called my colleague […]. Participant 11 –Health worker

Our observations and informal discussions with health professionals suggest that according to established practices in this context, pregnant women and women in labour are rarely accompanied by men. *“I rarely see men*, *and it’s because they don’t come*.*”* Participant 11 –Health worker

Those who come during the labour are not admitted into the delivery room. *“The men can’t come in*.*”* Participant 13 –Health worker.

Nevertheless, they play an important role during the pregnancy, because health workers involve them in helping to respect certain food restrictions to protect the parturient mother’s health.

Some of them, when they tell their husbands [what they’ve been told], their husbands don’t necessarily believe them. So sometimes they have to come back with their husband so that we can explain things to him. You see, the woman is bleeding. This is because of the heavy work. […] you have to let her rest. […] then the husband accepts and we give her an authorization to rest. Participant 10—Health worker

Finally, an oppressive marital relationship might affect the attitude of the woman in her interactions with health professionals.

Well, you know, what I described, it’s not fear, it’s the living environment itself…[…] of women who are subordinate within the home; really, when she comes here too, you can feel that she doesn’t like to talk. She doesn’t like to talk! It makes a difference […]. Participant 1 –Health worker

### Other challenges to the PCA

#### Health professionals continued training

Finally, continued training of health professionals is another key challenge. They have expressed a need to get training in PCA and to keep their professional knowledge up to date.

[…] Often you see people with information and you’re sitting there with your old knowledge, this doesn’t help. […] mostly with trainees; things change a lot and they come in with new knowledge. We are practicing, with old knowledge; sometimes you do something and they tell you no, this is how you do it, it has changed. It is often frustrating […]. Participant 7 –Health worker

And if this were taught in school? I think this too is a good idea, training school. If this could be added to medical school, it would help people. Participant 1 –Group discussion with parturient women

Generally, we don’t take into consideration people, the main actors, when things don’t work. If we involved them, it would be an improvement. We have to start raising awareness among them and it will work. We need to understand things and commit. […]. But without explaining, without a method, it’s going to take time. Participant 11 –Health worker

#### Collaboration with traditional practitioners

They also addressed interprofessional collaboration, including with traditional practitioners, which can sometimes facilitate implementing the PCA approach.

[…] some sick people, before they go to a hospital, first they see an indigénat, and they start treatment with tree bark at home. If their condition doesn’t improve, then they go to the hospital. Therefore, we need to involve them too, they will talk to us, share their experience. It can help. Patient 1 –Discussion group with health workers

These results are summarized in Table 4

**Table 4:** Summary of the main study findings by framework analysis component.

Component	Main themes and excerpts	Main challenges and excerpts
Biopsychosocial perspective	Pregnancy follow-up consultations cover more than physical health issues Before prenatal consultations, we have listening and discussion sessions. We talk about hygiene, family planning, and preparing for delivery. Participant 4 –Health worker	Addressing issues relating to sexual health in the presence of other women. Ah! As women, there may be something you want to ask, but you’re embarrassed. […] for example, when there is something in your genitals, you want to ask if there’s something but isn’t it embarrassing? It is embarrassing to ask! Participant 2 –Parturient woman
The professional as a person	Health professionals’ moods might affect their relationship with parturient women and the care they provide to the latter Sometimes you go there, and there’s a lot of work, or there has been a misunderstanding between them, they bug you and mistreat you when they talk to you. Participant 7 –Parturient woman […] sometimes you come and you’re so glad. Instead of a 10-minute discussion, you go on for 15 to 30 minutes and people find that you’re taking your time outside. Participant 4 –Health worker	Psychotherapy for Health Professionals We health workers also need a psychologist […] to whom we can talk about our problems. This can help us with mood management. Health worker–Discussion group with health professionals
The patient as a person	Parturient women asking to be well received, listened to, and respected Although you are sick, you come here and you’re respected, one takes care of you, it is really soothing. It gives you courage (laughs). Feeling welcome is also something. It makes you feel good. Participant 1- Group discussion with patients Above all respect and listening. Respect and listening. To care for patients, one must listen to them carefully. If the person is afraid…Fear, you know…if health workers could truly respect patients and listen to what they have to say, things would change. Regardless of who the person is, she must be respected and listened to. If you listen to her and you respect her now, the rest will follow. Participant 1- Discussion group with patients	Taking into account cultural and linguistic differences […] I often run into difficulty because I am not Mossi. I can’t explain exactly what I want to the women. I don’t understand Moré very well. Oftentimes, when we call in a third person to explain […] we’re not sure that this person will pass on exactly what you want to say. Participant 7 –Health worker […] certain [health workers] answer [my questions], but some don’t understand Moré. Participant 18 –Parturient woman
Power sharing, and rights and responsibilities of professionals and patients	Caregivers having to demonstrate their expertise Some are well informed and knowledgeable; they come at least to see us. In this case, you have to even prove your expertise that you are the health worker, that you are…you are the provider. You also have to prove to the patient that you were trained to provide care. Participant 10 –Health worker We don’t know anything about this…for me, midwives are the ones who should make decisions about childbirth. […] Anything they say is […] right. Participant 16 –Parturient woman	Raise awareness among patients about their rights and health professional’s responsibilities Patients don’t have the right to say anything over there. If you know this, you should have stayed home. If you come, you have to be careful with your words. Sometimes it is even better to stay silent and let them take care of things. Participant 1 –Discussion group with parturient women
Therapeutic alliance	Health professionals who are open to involving patients in decision-making regarding their care It works well when we try to involve the woman. She is the person concerned. So if we explain the dangers to her, it works. Care at least, works quite well. Participant 13 –Health worker Patients asking to have their say in service organization When you want to go, you have to get there early. If you go early, you’re seen rapidly, but if you go late and the number of people they’ve reached the number of people they can take in, you have to go back the following day. Participant 6 –Parturient woman You say no, I’ve come for the PNC. They’ll tell you “get up, you have to come back such other day”. So…And there are others, they’ll have to leave…3 km to get there. And if you go, you’re not taken, you have to go back again. Participant 1 –Group discussion with parturient women	Accepting birth attendants’ presence in the delivery room while avoiding traditional practices that are contrary to scientific recommendations For example, some older women, when you wait for the head to come out before you ease the baby out, they find that no, according to their practices, they push on the uterus to pushout the baby. We tell them no, this is not how it is done. They say that if you don’t do that, the baby will only come out a little or it won’t go through. And if by chance dystocia occurs and you have to refer, she’ll say that it’s because you don’t know how to do things, that you should push in order for the baby to come out. Participant 4 –Health worker
**Other PCA components**	Violence against patients in healthcare settings, tolerated and justified Well…Hitting there…or even hitting…It happens that we hit. When you’re alone, you have to get on with it. You’ve prepared everything, everything, you do this, you do that […] to be able to perform the delivery. And to your dismay, she crosses her legs. It can be systematic. You can yell at her, like you can hit her foot. This is what people are criticizing but […] as a health worker, I don’t see how this is bad. In a way, it’s to save a life that we do this […] because when a child dies, that too is a problem […]. Participant 8 –Health worker Involving men in care can contribute to protecting the mother and the child’s health Some of them, when they tell their husbands [what they’ve been told], their husbands don’t necessarily believe them. So sometimes they have to come back with their husband so that we can explain things to him. You see, the woman is bleeding. This is because of the heavy work. […] you have to let her rest. […] then the husband accepts and we give her an authorization to rest. Participant 10—Health worker	Health professionals’ continued training […] Often you see people with information and you’re sitting there with your old knowledge, this doesn’t help. […] mostly with trainees; things change a lot and they come in with new knowledge. We are in practice, with old knowledge; sometimes you do something and they tell you no, this is how you do it, it has changed. It is often frustrating […]. Participant 7 –Health worker

## Discussion

Our objective in this study was to understand the meaning attributed to the PCA in the context of Burkina Faso and to identify the challenges faced in the process of its adoption from the perspective of professionals and women using the services concerned.

With respect to the biopsychosocial component of the PCA, our results suggest that patients are provided holistic care, inasmuch as they are encouraged by health professionals to express their concerns regarding their health, the care received, as well as their everyday life. However, addressing in the presence of other women health issues, even though they are common to parturient women, in particular those relating to sexuality, appears to be a challenge. More conducive atmosphere for open discussions should therefore be implemented in that context of care. In fact as reported by Asafa et al [26]. in a study focusing on the challenges related to obstetric care access for women in rural Bangladesh, most rural women are intimidated when interacting with health professionals and are often fearful to express their feelings. In addition, they noted that women’s privacy is not well protected because of a lack of cultural understanding and irresponsible attitudes toward poor women [26]. Parturient women attach great importance to privacy protection [6]. Therefore, health professionals practicing in Burkina Faso should be sensitive and educated about the culture of the rural population in order to protect as much as possible women’s privacy in the context of consultations. These measures could help optimize the biopsychosocial component of PCA in maternal and child care.

As regards the physician or caregiver as a person aspect, our results indicate that their mood affects the care provided to parturient women. In fact, the latter expressed being often victim to verbal aggression on the part of caregivers, resulting from inner unrest that they experience in relation to family issues of their own or conflicts within the team. Such aggressive behaviour has been largely documented in low- and middle-income countries, associated with organizational problems such as the shortage of professionals, a high number of patients, low salaries, long work hours, and a lack of infrastructure, creating an environment conducive to poor professional conduct [6]. The contribution of our study in relation to this behaviour consists in shedding light on the impact of problems within the couple and the family, and within the caregiver team on the type of care and attention given to patients. Our study also highlights the importance of psychological support for caregivers to help them better deal with their personal issues and hence preclude the detrimental influence thereof on healthcare service users.

Concerning the patient as a person, our results confirm the need for patients to be well received, listened to, and respected in order to develop trustful relationships with caregivers and therefore achieve better health results. Nevertheless, taking into consideration cultural and linguistic differences continues to be a challenge as expressed by patients.

Regarding the aspect relating to power and responsibility sharing between patient and health professional, we found that professionals expressed expertise in care and concern for the patient’s input only in terms of service organization. There remain major challenges relating to the implementation of this component of the PCA including the disregard for the patients’ right to expression and for the professional’s duty to answer their questions. The concepts of superiority of healthcare personnel over patients, and entitlement to pass moral judgement and correct the patient are introduced by health training institutions, including the curriculum [27]. Consequently, change will require the involvement of all levels, in particular figures of authority in the healthcare profession and educators of future generations of caregivers [27].

Finally, as concerns therapeutic alliance, our findings indicate the importance of involving women patients not only in decision-making regarding their care, but also on the level of organizing health services. However, means for optimizing these alliances through deep partnerships with patients taking into account their backgrounds, socioeconomical status and religion could be included in the training of caregivers.

Our results indicate that men play an important role in maternity health, they help in the respect of treatment[28]. However, challenges related to the health structure, the lack of privacy, is notable. The use of digital health has a promising avenue with regards to the involvement of the men in the maternal health[29].

### Limitations of the study

Data was collected within an analysis framework. In so doing, interviews were structured in such a way as to collect precise information and therefore allowed the interviewee little leeway for exploring topics other than those suggested by the researchers. We sought agreement between coders, but it was not used for the purpose of topic selection, because this would have impacted the relevance of the chosen topics. This limitation opens avenues for future research, namely in order to take into account coder agreement in theme selection and results interpretation.

This analysis rests on the perspective of patients and health professionals from public healthcare facilities studied in the context of the MACHIP 2 project. We did not obtain the perspective of patients and professionals from other public and private health institutions that may differ from that of the groups represented in this study.

## Conclusion

A person-centred approach seems to be a familiar concept both in caregivers and patients interviewed in our study. However, adopting it on a routine basis is a challenge. There is more room for optimizing the implementation of PCA. For example, creating a more conducive environment for patients by refocussing caregiver training with regard for sensitivity to the cultural specificity of patient populations are among means that could be used. This may also entail organizational adjustments in order to create physical environments that are favourable to lending patients an attentive ear. Stakeholders at all levels are therefore concerned, in particular authority figures from various health professions (heads of professional orders, rectors of medical schools, directors of nursing and midwifery schools) and instructors (medical, nursing, and midwifery school professors). Training them in the principles of the patient-centred approach as planned for the second phase of this research, and educating them about the pertinence of this approach, are promising avenues.

## Supporting information

S1 File (DOCX)
