# Supplementary material for: Health professionals and patients’ perspectives on person-centred maternal and child healthcare in Burkina Faso
Source: PLoS One. 2020 Apr 1;15(4):e0230340. doi: 10.1371/journal.pone.0230340 (PMC7112215; doi:10.1371/journal.pone.0230340)
Supplement: S1 File — (DOCX) [file pone.0230340.s001.docx]

# **Annex 1.a. Individual interview guide (Participant)**

**
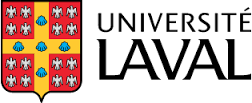
**

**« Transcultural validation of the person-centred approach in Koudougou, Burkina Faso »**

**Individual interview guide (Participant)**

**Record the verbal (audio) consent or have the written consent signed.**

**Welcome and Introduction : start the recorder.**

Thank you for agreeing to participate in this interview. My name is Thècle Twungubumwe. I am a student in the Master's program of Community Health with a major in Global Health at Laval University in Quebec City, under the supervision of Dr. Maman Joyce Dogba, a regular assistant professor at the Faculty of Medicine at Laval University. Today I would like to talk about the person-centred approach. This is a different way of providing care by discussing with patients, all the issues and decisions regarding their health status. By processing this way, the health care professional will take into account the values, needs, desires and preferences of patients. Patients seem more satisfied and adhere much more to treatments, which improves the effectiveness of care.

**Let the interpreter speak if necessary.**

I will start by asking you some questions about communication with the patient. Then, we will talk about cooperation between the health professional and the patient. The next step will focus on questions regarding health promotion. Finally, I'd like to ask you some questions on your social and demographic situation.

Please, note that there are no good or wrong answers.

**Let the interpreter speak if necessary.**

**Questions**

**Pregnancy :**

1. Hello, can you talk to me about your pregnancy ?
2. How is it going ?
3. Do you have any complications, any discomfort ?
4. Have you done your prenatal consultations (weighing) ? If yes, how many ?
5. Why ? If you haven’t done, the 4 recommended weighing ?
6. How are the weighing going ?
7. How were you received ?
8. Were you greeted ?
9. Are they concerned on your life ?
10. Is it important for you to be greeted and to have people concerned on your life ?
11. How do you know people are concerned in your life ?
12. Did you have someone accompany you during consultations ? If so, by whom and if not, why ?
13. Which recommendations did you received from the health care workers ?
14. Were your lifestyle habits taken into account when you received these recommendations ? E.g. : diet, physical effort, family context.
15. General speaking, what are the difficulties encountered regarding the follow-up of midwives' recommendations? E.g. : taking medication (iron), keeping appointments.
    1. Are your difficulties taken into account by the health workers ? If yes, how and if not, why ?
    2. Would you prefer your opinion to be taken into account ?
16. In your opinion, should decisions about pregnancy be made by the health worker ?

E.g., choosing between liquid or tablet iron, appointments.

- 1. Should you be included in this decision-making ?
  2. How can we ensure that you are included ?

1. What are your impressions on the care you’re receiving during your pregnancy ?

**Childbirth :**

1. Can you please talk to me about your childbirth ?
2. How were you received ?
3. Did you have someone accompany you ? If so, by whom and if not, why ?
4. Did you give a natural or caesarean birth ?
   - 1. Did you have a choice for the delivery method or was it imposed to you ?
5. Did you receive support from the health workers in attendance ?
6. Is this your first birth ? If not, how were the previous ones ?
7. How did you get to the CSPS, CM or CHR ? e.g. on foot, by bicycle, motorcycle, car.
8. How do you think an ideal delivery should take place ?
9. Is this achievable ?
10. In your opinion, what are the obstacles to this ideal delivery ?
11. In your opinion, how can these obstacles be overcome ?
12. In your opinion, should decisions about delivery be made by the health worker ? E.g. having someone accompany you during the delivery.
13. Should you be included in this decision-making ?
14. How can we ensure that you are included ?
15. What are your impressions about the care you received during your childbirth ?

**Postpartum :**

1. Since you gave birth, how are things at home ?
2. Feeding the baby
3. Bed time management
4. Receiving support from relatives at home
5. Can you talk to me about the follow-up on day 6 ?
   1. How were you received ?
   2. How was the weighing ?
   3. Are they interested in you since you gave birth ?
6. Can you talk to me about the follow-up on day 42 ?
   1. How were you received ?
   2. How was the weighing ?
   3. Are they interested in you since you gave birth ?
7. In your opinion, should decisions about your baby weighing be made by the health worker ?

E.g. Vaccination.

1. Should you be included in this decision-making ?
2. How can we ensure that you are included ?
3. What are your impressions about the care you received during the weighing sessions of your baby ?

**General questions :**

1. What are your general opinions on the services you received from midwives, nurses or birth attendants ?
   1. How were you received ?
   2. Were the behaviours satisfactory ?
   3. Were they able to answer your questions ? If not, why ?
2. Did you face a time when you had questions, concerns and were unable to ask them to the midwives, nurses or birth attendants ?
   1. Why ? E.g. fear, shame, shyness, etc.
3. Are you encouraged by midwives, nurses or birth attendants to ask your questions ?
   1. How do you think they should do it ?
4. Do you feel you've ever been mistreated or unwelcome ? E.g. People are sometime insulted or beaten during childbirth, being ridiculed because of the questions they ask or looked bad.
   1. If so, by whom, how, why ?
   2. How do you find this kind of behaviour? Does it shock you, doesn't bother you, you are used to it, is it normal ?
5. Talking on the way you are treated (Welcoming, choice of treatment [medication, delivery]), what do you think should be improved ?

**Suggestions and recommendations**

Finally, do you have any suggestions or comments to improve the training of health care professionals ?

Would you like to participate in a focus group ?

Thank you very much for your participation.

# **Annex 1.b. Individual interview guide (Health professional)**

**
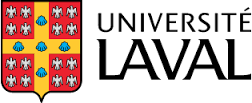
**

**« Transcultural validation of the person-centred approach in Koudougou, Burkina Faso »**

**Individual interview guide (Health professional)**

**Record the verbal (audio) consent or have the written consent signed.**

**Welcome and Introduction : start the recorder.**

Thank you for agreeing to participate in this interview. My name is Thècle Twungubumwe. I am a student in the Master's program of Community Health with a major in Global Health at Laval University in Quebec City, under the supervision of Dr. Maman Joyce Dogba, a regular assistant professor at the Faculty of Medicine at Laval University. Today I would like to talk about the person-centred approach. This is a different way of providing care by discussing with patients, all the issues and decisions regarding their health status. By processing this way, the health care professional will take into account the values, needs, desires and preferences of patients. Patients seem more satisfied and adhere much more to treatments, which improves the effectiveness of care.

I will start by asking you some questions about communication with the patient. Then, we will talk about cooperation between the health professional and the patient. The next step will focus on questions regarding health promotion. Finally, I'd like to ask you some questions on your social and demographic situation.

Please, note that there are no good or wrong answers.

**Questions**

**Hello,**

**Prenatal consultation (PNC) :**

1. Please, tell me first about yourself ?
   1. About your training ?
   2. Where do you work ?
   3. How long have you been working ?
   4. What kind of health professional are you ? (Doctor, nurse, midwife, birth attendant, AIS)
   5. How many years of experience do you have in this profession ?
2. Generally speaking, how is the medical consultations with pregnant women from the time they arrive to the time they leave ?
   1. How do you start the interview ?
      1. How do you welcome the woman ?
      2. Do you find time to check if she has any questions ?
         1. Does she ask questions when you give them the opportunity ? If yes, which questions come up most often and if not, why ?
3. When she asks questions, are you able to answer clearly and concisely ? (E.g. avoid using medical terms).
4. Do you think that lifestyle habits should be considered when giving care? (E.g. diet, family context etc.)
5. Can you please explain to me how you usually process when you provide care?
   1. What do you do first ?
   2. Do you talk with the woman while providing care ?
      1. Can voice her concerns or questions ?
      2. Do you ask her questions about the way she deals with the pregnancy at home ?
6. Before providing care, do you explain to the woman what you will be doing ? If so, how and if not, why ? (E.g., before taking her BP, before touching her body).
7. Did you notice some moment when it was impossible for you to explain to the woman what you were doing ?
   1. Can you please, give more details on what happened ?
8. How much time do you have for each consultation per woman ?
   - - - 1. In your opinion, is this time sufficient ?
         2. Does it allow you to give quality care ? If yes, why, if not, why not ?
9. Can you please, tell me about an event where your mood had a positive or negative impact on care ?
10. In your opinion, what level of expertise do you think a patient can have on her pregnancy? (E.g. low, medium, high)
11. How do you think the patient's knowledge can be taken into consideration for her treatment ?
12. What might be the barriers on using the patient's knowledge?
13. What can be the effects of involving the patient in the management of her pregnancy?
14. In your opinion, how would you describe a perfect consultation? (E.g. date of first PNC, how the consultation would proceed, etc.).

**Childbirth :**

1. Generally speaking, can you explain to me the process of delivery you usually apply from the time they arrive to the time they leave ?
   1. Who is in charge of the delivery ?
   2. How is the woman received ?
   3. Can she be accompanied in the delivery room ?
   4. What is your point of view on the presence in the delivery room, of someone accompanying the woman ?
2. What are the impacts on childbirth ?
3. While doing child birth, do you have the time to talk with the woman ?
4. If yes, what are your impressions ?
5. If not, what usually holds you back ?
6. Do you think it is important to talk with the woman during the delivery ?
7. How much time do you have for each consultation per woman ?
   - - - 1. In your opinion, is this time sufficient ?
         2. Does it allow you to give quality care ? If yes, why, if not, why not ?
8. I am also a nurse and I understand there is a lot of work, a lot of people to see and that you can get overworked; do you manage to find time to listen to patients' problems?
   1. How much time do you have for that ?
9. Do you find it important to take some time to listen to the patient ?
   1. If yes why, if not, why not ?
      1. If yes, how do you encourage the patient to ask more questions ?
10. How do you explain to the woman what you will be doing during childbirth ?
11. Can you please, tell me about an event where your mood had a positive or negative impact on the care ?
12. In your opinion, what level of expertise do you think a patient can have about her pregnancy? (E.g. low, medium, high)
13. How do you think the patient's knowledge can be taken into consideration for her treatment ?
14. What might be the barriers on using the patient's knowledge ?
15. What can be the effects of involving the patient in the management of her pregnancy?
16. In your opinion, how can we describe a perfect consultation? (E.g. date of first PNC, how the consultation would proceed, etc.).

**General questions :**

1. What are your general impressions on the services you usually provide ?
2. In your opinion, what impressions do women have on your services ?
3. I have heard of some behaviours, such as patients saying they had been yelled at, insulted or hit during deliveries, consultations, etc.
   1. What do you think of these behaviours ?
   2. What do you think should be done to reduce these behaviours ?

**This is part 2 : Patient-centred or person-centred approach.**

1. Patient-centred care is defined as care that responds to patients' individual values, needs, preferences and desires. It is an approach that allows care to revolve around the patient. This approach is showing good results in many countries : patients are more satisfied with health services and this approach increases the quality of care and the use of health services. We would like to develop it in Burkina Faso.

Here are the five basic principles

| **Socialbiopsycho perspective.** | **The patient as a person** | **Sharing power and responsibility between the Health professional and the patient.** | **The therapeutic alliance between caregiver and patient** | **The care giver as a person** |
| --- | --- | --- | --- | --- |
| Understanding the disease beyond the usual biomedical pathways | The patient is not simply defined as a body with a disease. | The care partnership : Empowering patients. | The partnership of care :  Addressing the complexity of patients' subjective experiences and supporting their autonomy and self-determination in managing their care. | He's not just a qualified technician. |

1. Is this feasible in Burkina Faso ? If it is not feasible, why not ?
2. How can we bypass the problems?

**Suggestions and recommendations**

Finally, do you have any suggestions or comments to improve the training of health professionals ?

Thank you very much for your participation.

# **Annex 1.c. Focus group interview guide (Participant)**

**
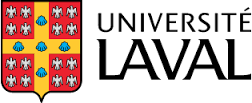
**

**« Transcultural validation of the person-centred approach in Koudougou, Burkina Faso »**

Focus Group interview guide (Participant)

Number of participants : _________

**Welcome and Introduction :**

Thank you for agreeing to participate in this interview. My name is Thècle Twungubumwe. I am a student in the Master's program of Community Health with a major in Global Health at Laval University in Quebec City, under the supervision of Dr. Maman Joyce Dogba, a regular assistant professor at the Faculty of Medicine at Laval University. Today I would like to talk about the person-centred approach. This is a different way of providing care by discussing with patients, all the issues and decisions regarding their health status. By processing this way, the health care professional will take into account the values, needs, desires and preferences of patients. Patients seem more satisfied and adhere much more to treatments, which improves the effectiveness of care.

We invite you to talk about any experience you face with a health professional (midwife, nurse, health worker, birth attendant, etc.) during your medical visits. We also hope to have your opinion on this new way of giving care in order to better educate health professionals. In all, we are planning five discussion groups of this type.

Please note that there are no good or wrong answers.

**Answer any questions participants may have.**

**Give the opportunity to anyone who does not want to participate to leave.**

**Ask those who want to participate to record the verbal (audio) consent or have the written consent signed.**

**Questions for the focus group (Attendee)**

**Start the recorder.**

1. First, let's get to know each other ! Tell me a little about yourself ?
   1. Your name, age ?
   2. Profession ?
   3. Child, how many and age ?
   4. Married ?

**Person-centred approach.**

1. Patient-centred care is defined as care that responds to patients' individual values, needs, preferences and desires. It is an approach that allows care to revolve around the patient. This approach is showing good results in many countries : patients are more satisfied with health services and this approach increases the quality of care and the use of health services. We would like to develop it in Burkina Faso.

Here are the five basic principles

| **Socialbiopsycho perspective.** | **The patient as a person** | **Sharing power and responsibility between the Health professional and the patient.** | **The therapeutic alliance between caregiver and patient** | **The care giver as a person** |
| --- | --- | --- | --- | --- |
| Understanding the disease beyond the usual biomedical pathways | The patient is not simply defined as a body with a disease. | The care partnership : Empowering patients. | The partnership of care :  Addressing the complexity of patients' subjective experiences and supporting their autonomy and self-determination in managing their care. | He's not just a qualified technician. |

1. Do you understand ?
2. In your opinion, do you think this approach is feasible, realistic?
3. What obstacles do you think we may encounter in implementing this approach ?

a) How do you think these obstacles can be overcome ?

1. We identified some barriers on implementing the person-centred approach, after the interviews we conducted
   1. The health worker is placed on a pedestal.
   2. Health workers' misperception of the person-centred approach. (The health worker sees PCA as a threat to his or her work.)
   3. Lack of knowledge of the user about her care (pregnancy, delivery, postpartum)
   4. Lack of human resources ? (To have time to talk with health workers)
   5. The ego of the health worker
2. How do you think these obstacles can be overcome ?
3. During your interactions with the health workers, did you feel that this approach was used ?
   1. If so, how, what has been done ?
   2. If not, what has not been done ?
   3. What would have facilitated this approach ?

**The partnership** :

The partner patient is a person who is progressively empowered, during his clinical journey, to make free and informed health choices. His/her experiential knowledge is recognized, and his/her care skills developed by the clinical team ^[[1]](#footnote-1)^.

The concept of care partnership is constantly evolving and is associated with a positive care experience for both the patient and the health-care professional. The partnership can be implemented from a democratic (recognized as a right), instrumental or utilitarian perspective for the purposes of health and expert imperatives. Its ability to empower patients has been increasingly emphasized since 2010. To this end, the partnership stresses the importance of considering the complexity of patients' subjective experiences and supporting their autonomy and self-determination in the management of their care, with the aim of improving the carer-patient relationship (23, 25).

Indeed, a well-designed and well-implemented partnership increase patient involvement in the planning and delivery of health services (19, 23) and is associated with increased patient satisfaction and adherence to treatment (45-47), efficient care (23, 26, 27), enhancement of patients' knowledge and experiential knowledge (48, 49), and improved safety, equity and ultimately patient outcomes (10, 19).

1. To what degree do you think patients can be involved ?
2. What are the barriers to this involvement ?
3. How can they be overcome ?

**Suggestions and recommendations :**

Finally, do you have any suggestions or comments to improve the training of health professionals ? Thank you very much for your participation.

# **Annex 1.d. Focus group interview guide (Health professional)**

**
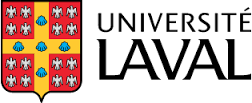
**

**« Transcultural validation of the person-centred approach in Koudougou, Burkina Faso »**

Focus Group Interview guide (Health professional)

Number of participants : _________

**Welcome and Introduction :**

Thank you for agreeing to participate in this interview. My name is Thècle Twungubumwe. I am a student in the Master's program of Community Health with a major in Global Health at Laval University in Quebec City, under the supervision of Dr. Maman Joyce Dogba, a regular assistant professor at the Faculty of Medicine at Laval University. Today I would like to talk about the person-centred approach. This is a different way of providing care by discussing with patients, all the issues and decisions regarding their health status. By processing this way, the health care professional will take into account the values, needs, desires and preferences of patients. Patients seem more satisfied and adhere much more to treatments, which improves the effectiveness of care.

Please note that there are no good or wrong answers.

We invite you to talk about any experience you face with a patient (female, male, pregnant or recently given birth, etc.) during his/her medical visit. We are also expecting to have your opinion on this new way of giving care in order to better educate health professionals. In a total, we are planning five discussion groups of this type.

Please note that there are no good or wrong answers.

**Answer any questions participants may have.**

**Give the opportunity to leave to any one who does not want to participate to leave.**

**Ask those who want to participate to record the verbal (audio) consent or have the written consent signed.**

**Questions for the focus group (Health professional)**

**Start the recorder.**

1. First, let's get to know each other ! Tell me a little about yourself ?
   1. Your name, age ?
   2. Profession ?
   3. Child, how many and age ?
   4. Married ?

**Person-centred approach.**

1. Patient-centred care is defined as care that responds to patients' individual values, needs, preferences and desires. It is an approach that allows care to revolve around the patient. This approach is showing good results in many countries : patients are more satisfied with health services and this approach increases the quality of care and the use of health services. We would like to develop it in Burkina Faso.

Here are the five basic principles

| **Socialbiopsycho perspective.** | **The patient as a person** | **Sharing power and responsibility between the Health professional and the patient.** | **The therapeutic alliance between caregiver and patient** | **The care giver as a person** |
| --- | --- | --- | --- | --- |
| Understanding the disease beyond the usual biomedical pathways | The patient is not simply defined as a body with a disease. | The care partnership : Empowering patients. | The partnership of care :  Addressing the complexity of patients' subjective experiences and supporting their autonomy and self-determination in managing their care. | He's not just a qualified technician. |

1. Do you understand ?
2. In your opinion, do you think this approach is feasible, realistic ?
3. What obstacles do you think we may encounter in implementing this approach ?

a) How do you think these obstacles can be overcome ?

1. We identified some barriers on implementing the person-centred approach, after the interviews we conducted :
   1. The health worker is placed on a pedestal.
   2. Health workers' misperception of the person-centred approach. (The health worker sees PCA as a threat to his or her work.)
   3. Lack of knowledge of the user about her care (pregnancy, delivery, postpartum)
   4. Lack of human resources ? (To have time to talk with health workers)
   5. The ego of the health worker
2. How do you think these obstacles can be overcome ?
3. During your interactions with the patients, did you have the impression you were using this approach ?
   1. If so, how, what has been done ?
   2. If not, what has not been done ?
   3. What could have facilitated this approach ?

**The partnership** :

The partner patient is a person who is progressively empowered, during his clinical journey, to make free and informed health choices. His/her experiential knowledge is recognized, and his/her care skills developed by the clinical team ^[[2]](#footnote-2)^.

The concept of care partnership is constantly evolving and is associated with a positive care experience for both the patient and the health-care professional. The partnership can be implemented from a democratic (recognized as a right), instrumental or utilitarian perspective for the purposes of health and expert imperatives. Its ability to empower patients has been increasingly emphasized since 2010. To this end, the partnership stresses the importance of considering the complexity of patients' subjective experiences and supporting their autonomy and self-determination in the management of their care, with the aim of improving the carer-patient relationship (23, 25).

Indeed, a well-designed and well-implemented partnership increase patient involvement in the planning and delivery of health services (19, 23) and is associated with increased patient satisfaction and adherence to treatment (45-47), efficient care (23, 26, 27), enhancement of patients' knowledge and experiential knowledge (48, 49), and improved safety, equity and ultimately patient outcomes (10, 19).

1. To what extent do you think patients can be involved ?
2. What are the barriers to this involvement ?
3. How can they be overcome ?

**The training :**

1. Tell me a little about the training you received ?
2. Usually, how are the training organized ?
3. How are people chosen to attend the training ?
4. Do you prefer an internal person or an external person to give you the training ?
5. What type of training do you prefer and why ? (Lecture, practical)
6. Once training has been completed, how can we ensure that the knowledge learned remains and is applied?

**Suggestions and recommendations :**

Finally, do you have any suggestions or comments to improve the training of health professionals ?

Thank you very much for your participation.

1. <http://www.ethique.gouv.qc.ca/fr/assets/documents/2013-ColloqueSSP-Acfas/Neron_Andre_Acfas%202013.pdf> [↑](#footnote-ref-1)
2. <http://www.ethique.gouv.qc.ca/fr/assets/documents/2013-ColloqueSSP-Acfas/Neron_Andre_Acfas%202013.pdf> [↑](#footnote-ref-2)
